# Notes and News

**Published:** 1888-10-27

**Authors:** 


### Notes and News.

Collected and Communicated.

Miss Lowther has left £1,000 to the Whitehaven Infirmary.

We are informed that a gold medal has been awarded to the Sanitas Company for its disinfectants, exhibited at the Ostend Health Exhibition.

Dr. and Mrs. Adams have been presented with a farewell address from the committee of the Cottage Hospital, Ashburton, on the occasion of their departure from that town.

Ten firms have sent in tenders for the erection of the proposed cottage hospital on Shooter's Hill, but the Woolwich Executive Committee have accepted none of them.

A new children's ward has been added to the Newport Infirmary. It is a large ward, prettily decorated, and contains fifteen cots.

The bust of the late Wilson Fox, M.D., F.R.S., will be unveiled by the Hon. W. H. B. Portman, at the Shire Hall, Taunton, on Thursday, October 25th, at 4.30 p.m.

Dr. Hekuys, an American physician attached to the American Mission at Ranipet, India, has died of hydrophobia. He was attacked by his own dog in church while holding a service.

Leicester Infirmary has a balance on the wrong side of no less than £2,000, and this with the fact that a children's hospital is just being completed under its shelter might not unnaturally give rise to serious anxiety. But Leicester knows no fear, and is now considering the formation of convalescent homes in the country, both for children and adults. May its faith in the generosity of its inhabitants be justified.

The Prince of Wales, accompanied by Lord Lytton, General Ellis, the Hon. Harry Tyrwhitt-Wilson, and Mr. Austin Lee, visited M. Pasteur's establishment in the Rue Vauclin last week, and saw several patients undergoing inoculation for rabies. Afterwards his Royal Highness went over the Pasteur Institute, which is nearly completed, and will be opened in a short time.

The new Eye Infirmary which has been in course of erection for some months at Wolverhampton, was opened on Tuesday last by the Earl of Dartmouth. The buildings consist of a main block for the in-patient department, with a wing next to Compton Road for the out-patients, attached to the main building, but with separate approaches. The in-patient department is a building three storeys high, in shape forming the letter E on the plan, with a main central entrance, and is designed on the corridor system. The whole of the work has involved an outlay of over £8,000.

Dr. Thelwall, of Farndon, has been presented with the Royal Humane Society's medal, for having rescued on August 28th, a man named Youde who had flung himself into the Dee, when it was in flood. Dr. Thelwall was passing, and at once jumped into the river and brought the man ashore in an unconscious condition.

It is sometimes our duty to condemn severely the expenditure at particular hospitals. There is no pleasure in that. But when a report reaches us which shows a minimum of expenditure with no lessening of efficiency it is a source of sincere gratification. The one conspicuous feature of the recently-issued report of the Stockton-on-Tees Hospital is the smallness of the cost per occupied bed per annum. A trifle over £35 is the amount. This is less than a quarter of the sum spent by some hospitals. A valuable table is printed with the report, which shows to the minutest detail how the sum is made up. It also gives comparative statements for the past seven years. In 1884 the daily cost per occupied bed was a little over 4*s.* 7½*d.* In 1888 the daily cost has fallen to a little under 2*s.* Since the former date the reduction has been gradual, but sure. It would be most interesting to know how it has been brought about. The secretary of the hospital is Mr. John Settle and the matron Miss Edith Lock.

A harvest thanksgiving service was held in the chapel at the Norfolk and Norwich Hospital on Sunday, October 14th. In the evening, when there was a large congregation, the service was conducted by the Chaplain (the Rev. G. Willoughby Barrett), the special preacher being the Rev. Canon Nisbet. The cathedral organist (Dr. Bates) presided at the organ, and eight boys from the cathedral choir assisted the nurses in rendering the musical portion of the service. The offertories in the chapel and the collections in the wards amounted to a total sum of £2 9*s.* 3*d.*, devoted to the general fund of the hospital. The chapel was very prettily decorated by the Lady Superintendent (Miss Adam) and the members of the nursing staff. At the foot of the cross on the communion table was placed a loaf of bread, surrounded by fine bunches of grapes. Miniature sheaves of wheat and vases of dahlias were tastefully arranged on either side of the cross. The sills in the east windows were filled with fruit, and at each end of the reredos was a large sheaf of wheat. Wheat straw, interspersed with natural grasses, ivy tendrils, etc., adorned the chancel arch, and the low chancel screen was bedecked with oak leaves, hedge-row berries, and small baskets of fruit.

Those who are following our series of articles on "Voyages in Vacation" may be glad to know that the Orient Line have planned an autumn tour in the Mediterranean in their steamship *Garonne*, 3,000 horse-power, under the charge of Commander W. E. White, R.N.R. The *Garonne* is timed to leave London on the 15th November, and is due back there on the 22nd December. The tour includes a visit to Lisbon, Gibraltar, Algiers, Palermo, Naples, Rome, Genoa, Nice, and the Alhambra. Early application should be made for berths, as the number of passengers is strictly limited. Some who prefer to take a more extended tour may wish to take advantage of the opportunity afforded by the well-known Peninsular and Oriental Steam Navigation Company, whose steamers on their Eastern voyages touch at the islands of the Mediterranean, Gibraltar, and Malta; at Marseilles, whence the Riviera or Algeria can be best reached; at Naples, for Southern Italy; and at Alexandria, Port Said, and Ismalia, for Egypt and the Nile. The entire change of scene and mode of life, the cheerful society and bracing air—in short, all the pleasures and advantages of a yachting cruise, without its discomforts—will, no doubt, render these trips fashionable with those whose means and leisure enable them to exchange the rigors of our winter for the sunny climes of the South.

THE following vacancies are announced this week, the amount of salary in each case being given after the name of the institution :—

**Resident Medical Officers.**

Bootle Borough Hospital. House Surgeon.—£80, board, etc.
Wilts County Asylum. Second Assistant Medical Officer.– £100, with board, etc.
Wrexham Infirmary and Dispensary. House Surgeon.—£100, and lodging, gas, attendance.

**Non-resident Medical Officers.**

Chelsea Hospital for Women. Pathologist.
Dental Hospital of London, Leicester Square. Lecturer on Mechanical Dentistry.
Owens College, Manchester. Junior Demonstrator in Anatomy. —£100.

---

A "DOCTOR OF MEDICINE" has been writing an article for the *Newcastle Leader* on the subject of fever and fever hospitals, in which he wisely points out that such institutions benefit not only the sufferers but the mass of the population. He also remarks on the curious fact that infectious diseases which have been long prevalent in the population of a place tend to become less virulent. In reference to this a gentleman said the other day that when he was a boy he lived at Amersham, in Bucks. Scarlet fever was at that time always rife there, but nobody paid any heed to it. There was no isolation, no disinfection, and seldom a doctor called in to attend the patient. Certainly this gentleman could never remember a death caused by the fever, nor did any of the villagers ever seem to imagine for a moment that it was a dangerous disease. It caused no more sensation than a cold in the head, and did no more harm.

---

A CIRCULAR has been issued by the committee of management appointed for carrying out the work connected with the establishment of a cottage hospital in Falkirk, who, after considering the matter very fully, are of the opinion that the sum subscribed is inadequate for what will be required to meet the wants of the town and district. Revised plans, arranged with the view of carrying out the work as economically as possible, have been prepared by Messrs. A. and W. Black, architects. In addition to utilizing the present cottage for the accommodation of attendants, etc., it is intended to add a building with two wards, one each for males and females—each ward to contain eight beds, and having all necessary conveniences, bath rooms, etc. It is expected that the cost of the above, with the requisite outside offices, fittings, general furnishings, etc., and including the price of Saltoun Cottage and ground, which has been acquired for the purpose, will amount to about £1,300.

---

AN entirely new phase in the development and usefulness of an excellent institution—the Salford and Pendleton Royal Hospital—has just been entered upon, thanks to sundry large bequests by various benefactors, among whom may be noted the late John Pendlebury, of Pendleton, Alderman Rose, J. Worrall Walker, and Sir Joseph Whitworth. Until lately its capabilities have been very limited, but recently a fine addition has been made to the building at a cost of £12,000, which has been completed without the necessity of calling upon the inhabitants for any contribution whatever, thanks mainly to the generous bequest of Mr. Pendlebury. In its earliest days the institution was intended for a dispensary merely, but it has gradually grown until now there are two efficient dispensaries and a hospital containing beds for medical and surgical patients to the number of one hundred and twenty. Of these, forty are medical, eight are isolated for infectious and dangerous cases, and the remainder are surgical. The hospital has suffered a severe loss in the resignation of Mr. Nathaniel Shelmerdine, for forty years chairman and treasurer. Mr. Lamouth, the late secretary, has also resigned, and Mr. Alexander Hay has been appointed to the post.

THE opening of the new wards of Providence Free Hospital, St. Helens, which have been erected at a cost of over £2,000, afford an opportunity of briefly referring to the work done by this institution. Some few years ago a congregation of "sisters," under the leadership of Miss Taylor—who herself had been a fellow-worker with Miss Florence Nightingale in the hospitals of Scutari—was founded at Brentford. Half-a-dozen years ago a branch of the Order was opened in George Street, St. Helens. The sisterhood commenced in a very humble way, doing laundry work for a subsistence, and in their spare hours visiting among the poor and motherless, and attending the sick members of indigent families, by degrees, as means permitted, taking children and stricken women to their George Street Home for rest and nursing. Quickly the place became too small, and Miss Taylor, with a noble, womanly courage and a firm trust in Providence, ventured upon taking Hardshaw Hall, and happily designated it Providence Free Hospital. With the rapid growth of the borough this hospital was soon overcrowded, and Mrs. Peter Middlehurst nobly gave £2,000 to build two new wards to hold sixty beds. The hospital is strictly unsectarian.

---

FAITH in fish as an unequalled "brain food" is neither so universal nor so profound as it was a few years ago; but though that is so, no man of any authority has ventured to deny its general value as an article of dictary. A "Leeds Man" writes to a local paper asking why this excellent food is so little used in hospitals. He says that in many hospitals very little fish is eaten, whilst in some it is quite unknown at the table. He further affirms that fish is much cheaper than meat, and suggests two "fish days" a-week for hospital patients. If fish is cheap in Leeds that town is more fortunate than London. So confident are we of the value of it as an article of diet for invalids that we would gladly endorse all that is said by the Leeds correspondent, even to the extent of advocating two fish days a-week, if it were not for the question of cost. In the metropolis fish is so dear as to be practically unavailable for all persons of moderate means. We are under the impression that a very similar condition of things exists in most other large towns. If the "Leeds Man" really desires to confer a benefit on the community, and to earn the undying gratitude of all lovers of fish, he will organise a crusade in favour of cheap fish immediately.

---

THE meeting of the National Pension Fund, recently held at St. Thomas's Hospital, has quickened curiosity and interest in a degree which is altogether remarkable. We are informed that among other correspondents a "Nurse of Long Experience," who writes anonymously, expresses the conviction that nurses do not live so long on an average as other people, and she founds on this a claim for special consideration for those nurses who have seen long service. We quite agree with the anonymous correspondent that long service should entitle to special consideration, and the actuary himself does not object to this. We know the managers of the Fund desire to be as benevolent as possible, the Fund having been founded with the sole object of helping the nurses to the utmost. The managers want two things; first, to persuade nurses to help themselves, and then to help those who try to help themselves by means of large bonus additions to pensions. All nurses of every kind who anticipate difficulties and anxieties as age advances, should communicate with the manager, 8, King Street, E.C., giving their full name and address, and at least give him the opportunity of helping them. The whole rules of the Fund are drafted with the view of benefiting the pensioner. The pensioner has the first consideration of the council, and the longer the service the greater proportionately will be the resulting pension. As has been stated repeatedly, the whole of the profits from every source will be added to pensions, and the longest service will entitle to the largest claim.

